# HIV-Associated Neurocognitive Disorder and CNS Viral Escape: A Case Report

**DOI:** 10.7759/cureus.66992

**Published:** 2024-08-16

**Authors:** Keesari Sai Sandeep Reddy, Vijay B Reddy, Sai Teja Gummadi, Gunasekaran Nallusamy

**Affiliations:** 1 Internal Medicine, Saveetha Medical College and Hospital, Saveetha Institute of Medical and Technical Sciences, Saveetha University, Chennai, IND; 2 Internal Medicine, Apollo Insitute of Medical Sciences and Research, Hyderabad, IND

**Keywords:** cognitive impairment, antiretroviral therapy, cns viral escape, hand, hiv-associated neurocognitive disorder

## Abstract

HIV-associated neurocognitive disorder (HAND) encompasses a spectrum of cognitive impairments prevalent in individuals infected with HIV, despite effective combination antiretroviral therapy. This case report discusses a 42-year-old male with a history of HIV infection since 2014 who is currently on antiretroviral therapy (ART). The patient presented with cognitive impairment and generalized weakness, with subsequent investigations revealing HAND and CNS viral escape. The patient’s management involved a switch to a different ART regimen, resulting in significant clinical improvement. This case highlights the importance of considering CNS-specific HIV replication in patients with neurocognitive symptoms and underscores the need for tailored ART regimens. The exclusion of progressive multifocal leukoencephalopathy through negative JC virus PCR was crucial in directing appropriate management. Comprehensive diagnostic evaluations, including CSF analysis and brain imaging, are essential for the accurate diagnosis and effective treatment of HAND, particularly in cases of CNS viral escape.

## Introduction

HIV-associated neurocognitive disorder (HAND) represents a spectrum of cognitive impairments that occur in patients infected with HIV. Despite the advent and widespread use of combination antiretroviral therapy (cART), which has significantly reduced the morbidity and mortality associated with HIV infection, HAND continues to affect a substantial proportion of individuals living with HIV. It is estimated that up to 50% of HIV-infected individuals experience some degree of neurocognitive dysfunction during the course of their illness [1].

The pathogenesis of HAND is multifactorial, involving direct viral effects on the CNS, chronic inflammation, immune activation, and neuronal injury [2]. HIV can cross the blood-brain barrier early in the course of infection and establish a reservoir in the CNS, which poses a challenge for complete viral eradication [3]. Moreover, the CNS can serve as a sanctuary site where the virus persists despite systemic viral suppression, leading to what is known as CNS viral escape [4].

CNS viral escape is characterized by the detection of HIV RNA in the CSF in the presence of undetectable plasma HIV RNA levels. This phenomenon suggests that the virus can replicate independently in the CNS, contributing to neurocognitive decline even in patients receiving effective systemic cART [5]. The clinical presentation of HAND varies widely, ranging from asymptomatic neurocognitive impairment to HIV-associated dementia, with symptoms including memory loss, decreased attention, slowed psychomotor speed, and executive dysfunction [6].

MRI findings in patients with HAND often reveal diffuse white matter changes, predominantly in the frontal and parietal lobes, which correlate with the extent of cognitive impairment [7]. CSF analysis can provide critical insights into the viral dynamics within the CNS and guide therapeutic decision-making.

The significance of reporting this case lies in the detailed illustration of CNS viral escape and its impact on neurocognitive function, despite systemic viral suppression. This case underscores the need for clinicians to remain vigilant for symptoms of HAND and to consider CNS viral escape in the differential diagnosis, even in patients with controlled systemic HIV infection. Furthermore, it highlights the importance of timely and appropriate modifications in ART regimens to address CNS-specific viral replication, leading to significant clinical improvement, as observed in this patient.

In this case report, we present a 42-year-old male with a long-standing HIV infection who developed HAND and exhibited CNS viral escape. We discuss the diagnostic challenges, management strategies, and the patient’s clinical course following the modification of his antiretroviral regimen.

## Case presentation

A 42-year-old male patient had been diagnosed with HIV in 2014 and had been on ART since then. His ART regimen consisted of tenofovir-emtricitabine and atazanavir-ritonavir, which had kept his plasma viral load undetectable for several years. However, over the past few months, he began experiencing cognitive decline, which progressively worsened. He struggled with daily activities and work responsibilities due to his cognitive symptoms. He described his symptoms as slowness in activities, significant memory loss, and marked difficulty in concentrating, which affected his ability to perform routine tasks efficiently.

On examination, the patient was alert and cooperative but exhibited clear signs of cognitive impairment. His attention and calculation abilities were notably impaired, and his speech was slow, with decreased word output. A neurological examination revealed an MMSE score of 23, indicating mild cognitive impairment. Muscle strength testing revealed a strength of 4/5 in all four limbs, suggesting mild generalized weakness.

Laboratory tests revealed a CD4 lymphocyte count of 352 cells/µL and a plasma viral RNA level of <66.9 copies/mL, indicating effective systemic viral suppression. Complete blood counts, liver function tests, renal function tests, and serum electrolytes were all within normal ranges, ruling out common metabolic causes of cognitive impairment and generalized weakness.

**Figure 1:**
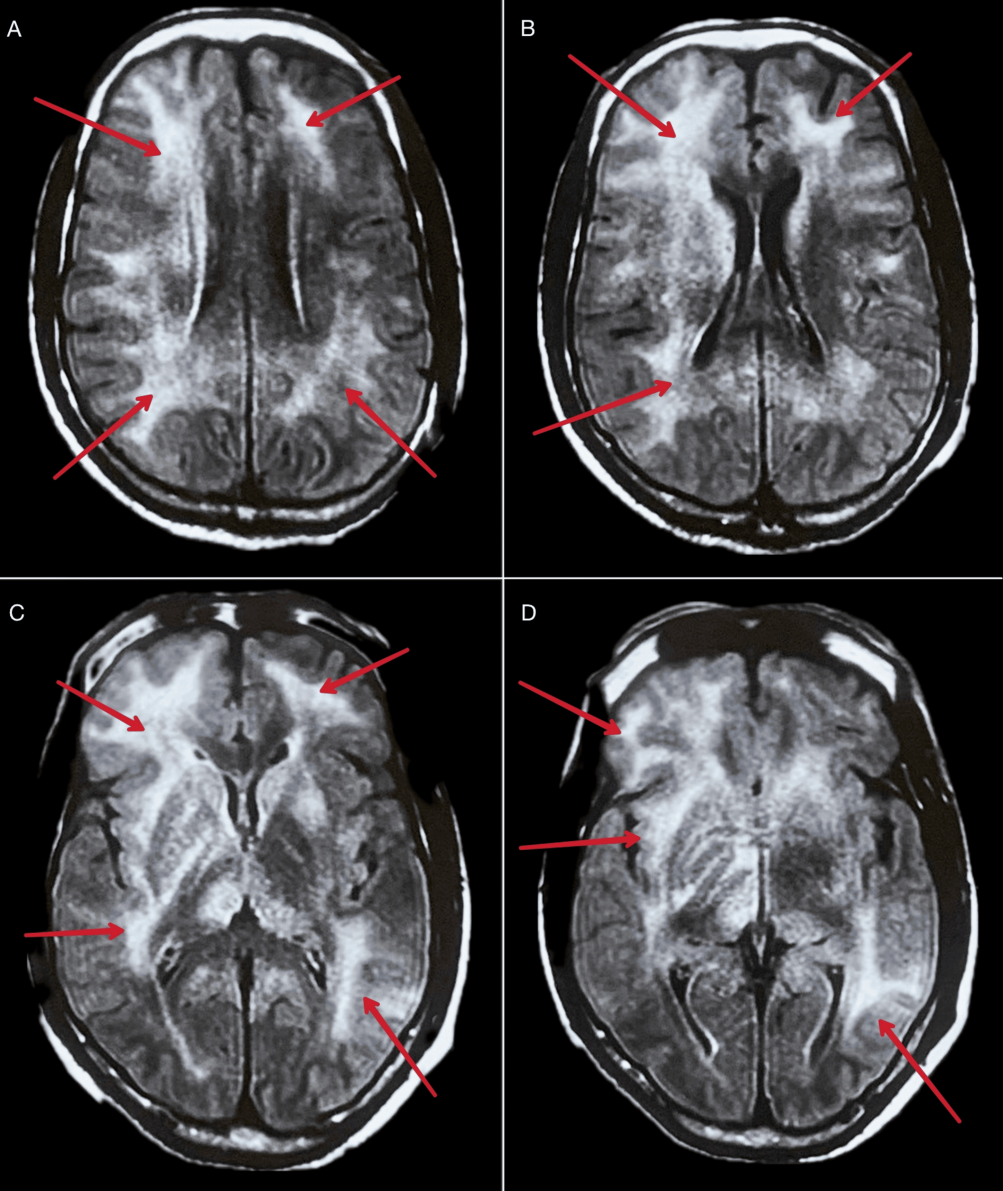
Brain MRI images, marked with red arrows, demonstrate diffuse white matter hyperintensities across multiple regions on FLAIR sequences. Panels A and B show hyperintensities in the bilateral frontal regions; panels C and D highlight similar findings in the temporal regions; and panels B, C, and D reveal the involvement of the parietal periventricular regions.

Brain MRI revealed diffuse bilateral frontal, temporal, and parietal periventricular white matter hyperintensities on T2-weighted and FLAIR sequences (Figure 1). These MRI findings were consistent with diffuse white matter changes often seen in HAND. CSF analysis indicated a viral load of 2,365 copies/mL, despite an undetectable plasma viral load, suggesting CNS viral escape. The JC virus PCR was negative.

Differential diagnosis

Progressive Multifocal Leukoencephalopathy (PML)

PML is a demyelinating disease caused by the reactivation of the JC virus in immunocompromised individuals, including those with HIV. The clinical presentation of PML can overlap with HAND, making it a critical consideration. PML often presents with focal neurological deficits, cognitive decline, and white matter changes on MRI. In this case, the negative JC virus PCR in the CSF helped exclude PML as a cause of the patient’s symptoms.

CNS Opportunistic Infections

Opportunistic infections such as toxoplasmosis, cryptococcal meningitis, and cytomegalovirus encephalitis can occur in HIV-infected individuals and present with neurological symptoms. However, these infections were less likely in this patient due to the absence of focal neurological signs, systemic symptoms, and negative findings for other pathogens in the CSF analysis.

Other Neurodegenerative Disorders

Conditions such as Alzheimer’s disease, frontotemporal dementia, and vascular dementia can present with cognitive decline. However, these disorders were less likely given the patient's history of HIV and the MRI findings. Additionally, the relatively rapid progression of symptoms and the specific pattern of white matter changes were more consistent with HAND.

HAND

HAND is characterized by cognitive impairment, motor dysfunction, and behavioral changes in patients with HIV. The patient’s history of HIV, along with his cognitive symptoms, strongly suggested HAND. This diagnosis was further supported by the MRI findings of diffuse white matter changes, which are typical of HAND.

Management

Based on the diagnosis of HAND and CNS viral escape, the patient’s ART regimen was switched to zidovudine, lamivudine, and dolutegravir to improve CNS penetration. This change aimed to more effectively control HIV replication within the CNS. The anticipated outcome was stabilization or improvement in cognitive function and overall neurological status with the new regimen due to its enhanced CNS penetration.

Follow-up

At the six-month follow-up, the patient demonstrated significant clinical improvement. His cognitive function improved markedly, allowing him to resume his job and daily activities. Repeat MRI showed stability of the white matter changes, and the CSF viral load decreased substantially, indicating a positive response to the new ART regimen.

## Discussion

HAND remains a significant clinical challenge despite advancements in cART. This case highlights the complexities of HAND, particularly in the context of CNS viral escape, where HIV replication persists in the CNS despite undetectable plasma viral loads.

The patient’s presentation with cognitive impairment, including slowness in activities, memory loss, and generalized weakness, is consistent with HAND. His MRI findings of diffuse bilateral frontal, temporal, and parietal periventricular white matter hyperintensities further supported this diagnosis. White matter changes in HAND are well documented and often correlate with cognitive deficits, as seen in this patient [8].

CSF analysis played a crucial role in this case. The elevated CSF viral load of 2,365 copies/mL, despite an undetectable plasma viral load, indicated CNS viral escape. CNS viral escape occurs when HIV replicates independently within the CNS, leading to localized inflammation and neurocognitive decline [9]. This phenomenon underscores the importance of evaluating CSF viral loads in patients with suspected HAND, even when systemic HIV parameters appear controlled.

The diagnosis of HAND with CNS viral escape necessitated a modification of the patient’s ART regimen. The initial regimen of tenofovir-emtricitabine and atazanavir-ritonavir was not sufficiently penetrating the CNS. The new regimen, consisting of zidovudine, lamivudine, and dolutegravir, was chosen for its better CNS penetration and efficacy against CNS HIV replication. Zidovudine and lamivudine are nucleoside reverse transcriptase inhibitors known for their ability to cross the blood-brain barrier, while dolutegravir is an integrase strand transfer inhibitor with potent activity against HIV [10,11]. This tailored approach resulted in significant clinical improvement, with the patient showing marked cognitive recovery and the ability to return to work within six months of the regimen change.

An important differential diagnosis in HIV-infected patients presenting with neurocognitive symptoms is PML, caused by reactivation of the JC virus. PML is a severe demyelinating disease that can occur in immunocompromised individuals, including those with HIV. The clinical presentation of PML can overlap with HAND, making it a critical consideration. In this case, the negative JC virus PCR in the CSF was significant as it helped exclude PML as a cause of the patient’s symptoms. This finding directed the focus toward managing HAND and CNS viral escape rather than initiating treatments for JC virus infection [12,13].

This case illustrates several key points in the management of HAND. First, clinicians must maintain a high index of suspicion for HAND in HIV-infected patients presenting with cognitive symptoms, even when systemic viral loads are suppressed. Second, CNS viral escape should be considered in the differential diagnosis, and CSF analysis should be performed to assess CNS viral loads. Third, ART regimens may need to be modified to include agents with better CNS penetration to effectively manage HAND and achieve clinical improvement [14].

The significance of this case lies in its detailed illustration of the diagnostic and therapeutic challenges associated with HAND and CNS viral escape. Current studies are exploring novel therapeutic approaches, including the use of adjunctive therapies such as anti-inflammatory agents and neuroprotective drugs to mitigate the impact of HAND [15].

## Conclusions

This case underscores the complexities involved in managing HAND, particularly in the context of CNS viral escape. Despite effective systemic antiretroviral therapy, the patient developed significant cognitive impairments due to localized CNS HIV replication. The marked clinical improvement following the modification of the ART regimen to include drugs with better CNS penetration highlights the importance of tailored treatment strategies in managing HAND. This case emphasizes the need for clinicians to remain vigilant for symptoms of HAND and to consider CNS viral escape in the differential diagnosis, even in patients with controlled systemic HIV infection. Comprehensive diagnostic evaluations, including CSF analysis and brain imaging, are crucial for accurate diagnosis and effective management. Further research is essential to understand the mechanisms underlying CNS viral escape and to develop more effective therapeutic approaches for HAND, ultimately improving the quality of life for HIV-infected individuals.
